# Activated CD90/Thy-1 fibroblasts co-express the Δ133p53β isoform and are associated with highly inflamed rheumatoid arthritis

**DOI:** 10.1186/s13075-023-03040-8

**Published:** 2023-04-15

**Authors:** Anna K. Wiles, Sunali Mehta, Melanie Millier, Adele G. Woolley, Kunyu Li, Kim Parker, Marina Kazantseva, Michelle Wilson, Katie Young, Sarah Bowie, Sankalita Ray, Tania L. Slatter, Lisa K. Stamp, Paul A. Hessian, Antony W. Braithwaite

**Affiliations:** 1 Department of Pathology, University of Otago, Hercus Building, 58 Hanover Street, Dunedin, New Zealand; 2 Maurice Wilkins Centre for Biodiscovery, University of Otago, Dunedin, New Zealand; 3 Department of Medicine, University of Otago, Dunedin, New Zealand; 4 Department of Medicine, University of Otago, Christchurch, New Zealand; 5 Malaghan Institute of Medical Research, PO Box 7060, Wellington, New Zealand

**Keywords:** p53 isoforms, Rheumatoid arthritis, Inflammation, Synoviocytes, Fibroblasts, CD90

## Abstract

**Background:**

The p53 isoform Δ133p53β is known to be associated with cancers driven by inflammation. Many of the features associated with the development of inflammation in rheumatoid arthritis (RA) parallel those evident in cancer progression. However, the role of this isoform in RA has not yet been explored. The aim of this study was to determine whether Δ133p53β is driving aggressive disease in RA.

**Methods:**

Using RA patient synovia, we carried out RT-qPCR and RNAScope-ISH to determine both protein and mRNA levels of Δ133p53 and p53. We also used IHC to determine the location and type of cells with elevated levels of Δ133p53β. Plasma cytokines were also measured using a BioPlex cytokine panel and data analysed by the Milliplex Analyst software.

**Results:**

Elevated levels of pro-inflammatory plasma cytokines were associated with synovia from RA patients displaying extensive tissue inflammation, increased immune cell infiltration and the highest levels of *Δ133TP53* and *TP53β* mRNA. Located in perivascular regions of synovial sub-lining and surrounding ectopic lymphoid structures (ELS) were a subset of cells with high levels of CD90, a marker of ‘activated fibroblasts’ together with elevated levels of Δ133p53β.

**Conclusions:**

Induction of Δ133p53β in CD90^+^ synovial fibroblasts leads to an increase in cytokine and chemokine expression and the recruitment of proinflammatory cells into the synovial joint, creating a persistently inflamed environment. Our results show that dysregulated expression of Δ133p53β could represent one of the early triggers in the immunopathogenesis of RA and actively perpetuates chronic synovial inflammation. Therefore, Δ133p53β could be used as a biomarker to identify RA patients more likely to develop aggressive disease who might benefit from targeted therapy to cytokines such as IL-6.

**Supplementary Information:**

The online version contains supplementary material available at 10.1186/s13075-023-03040-8.

## Background

Rheumatoid arthritis (RA) is a systemic autoimmune disease characterised by chronic inflammation of joint synovial tissue, resulting in destructive, debilitating disease with recognised systemic involvement [1]. Its aetiology is likely multifactorial, with a combination of environmental (toxins, lifestyle, epigenetic) [2, 3] and biological (viral, hormonal, microbiome) factors [4, 5], which initiate development in those genetically susceptible [6]. Classic pathological hallmarks of RA include synovial proliferation, apoptosis resistance, increasing angiogenesis and immune dysregulation. The underlying disturbance in immune regulation [7] is responsible for the infiltration of joint synovium by myeloid cells and T and B lymphocytes that become varyingly organised and display persistent aggregation and ectopic lymphoid structures (ELS) contributing cytokines and chemokines and promoting auto-antibody production [8–13]. Intriguingly, many of the features that accompany the development of inflammation in RA synovia parallel those evident in cancer progression including proliferation, immune dysregulation, cellular migration, invasion and metastasis to distant sites [14, 15].

Such tumour-like behaviour, when considered together with reduced apoptosis and hyperplasia, is consistent with impaired function of the tumour suppressor gene and critical cell cycle checkpoint regulator, *TP53*. Typically, mutations in the *TP53* gene, or aberrations in molecular pathways affecting p53 protein expression and regulation, lead to unchecked proliferation and tumour growth [16]. Elevated expression of murine double-minute protein 2 (MDM2), a major negative regulator of p53, is evident in synovial lining of cells in RA [17], and *TP53* missense mutations have been documented in intimal and sub-lining regions of RA synovial tissue representing the clonal expansion of cells [18], and these are associated with increased levels of the pro-inflammatory cytokine interleukin-6 (IL-6) [19]. Further, in vitro studies show that inhibition of wild-type (wt) p53 function increases the proliferation and invasiveness of RA synovial fibroblasts and also transforms normal fibroblasts to display aggressive behaviour similar to their rheumatoid counterparts [20]. While these studies are consistent with the suggestion that disruption of *TP53* plays a functional role in RA pathology, here we consider the possibility that dysregulated expression of p53 isoforms represents one of the early triggers in the immuno-pathogenesis of RA and actively perpetuates chronic synovial inflammation.

The *TP53* gene encodes at least 12 isoforms generated by a combination of alternative promoter usage, translational start sites and splicing [21–23]. This results in 4 isoform families: full-length p53 (FLp53) and the truncated versions, Δ40p53, Δ133p53 and Δ160p53 lacking 39, 132 and 159 amino acids at the N-terminus, respectively (Additional file 1: Fig. S1). Each of these families can encode 3 C-terminal alternatively spliced variants, designated by α, β or γ (Additional file 1: Fig. S1). Several of the isoforms have been shown to moderate p53 activities [21–23] as well as having unique independent functions. Elevated levels of *Δ133TP53* isoform have been reported for several cancers, and in breast, colorectal and prostate cancers, elevated *Δ133TP53β* mRNA is associated with reduced disease-free survival [24–26]. Of importance, the cancers with high levels of Δ133p53 isoforms are frequently associated with extensive immune cell infiltration [26, 27], suggesting Δ133p53 isoforms play an active role in recruiting immune cells to the tumour. To this end, Δ133p53 isoforms have been shown to directly increase the expression of multiple cytokine-related genes [25, 28]. Moreover, a transgenic mouse with a truncated *Trp53* gene that generates a ‘Δ133p53-like’ protein, designated Δ122p53 [29], shows profound inflammatory phenotypes, including lymphoid aggregates in multiple organs, prominent Peyer’s patches in the colon, elevated serum cytokine levels especially IL-6, vasculitis and production of auto-antibodies [29, 30].

Studies such as these emphasise the role of the Δ133p53 isoforms as immune system modulators. To investigate whether these isoforms play a role in promoting an auto-immune disease, we analysed synovial joint tissue from people with RA. The results showed that synovium from RA patients displaying extensive tissue inflammation had the highest levels of *Δ133TP53* and *TP53β* mRNA. Immunostaining identified a subset of cells that stain strongly for Δ133p53β isoform and co-express CD90, a marker of ‘activated fibroblasts’ [31]. We found these fibroblasts predominantly in the perivascular regions of the synovial sub-lining, around ELS. We also found elevated levels of pro-inflammatory circulating serum cytokines in people with RA displaying higher levels of synovial *Δ133TP53* and *TP53β* mRNA together with increased immune cell infiltration in the synovial membrane.

## Methods

### Patient cohort

Synovial tissue samples from RA or osteoarthritis (OA) patients undergoing joint replacement were used. Written informed consent was obtained from all participants. Clinical and demographic details of the participants are summarised in Table 1.

**Table 1:** Clinical and demographic characteristics of the rheumatoid arthritis and osteoarthritis cohorts

	Rheumatoid arthritis	Osteoarthritis
Patients (no.)	37^a^	20
Synovia	40	20
Female, *n* (%)	30 (82)	14 (70)
Age, years (mean ± SE)	62 ± 2	71 ± 2
Disease duration, years	17 ± 2	12 ± 3
RF positive (%)^b^	90	n.d
CCP^+^ (%)^b^	84	n.d
CRP^+^ (mg/dL)	10 ± 2	n.d
Radiographic erosions^d^	88	n.d
Subcutaneous nodules^d^	56	n.d
Disease-modifying anti-rheumatic drugs [no. (%)]		
Prednisone	9 (36)	–
bDMARDs	3 (12)	–
cDMARDs	10 (40)	–

For several variables, there was missing data in ≥ 1 of the patients

*bDMARD*, biological DMARD; *cDMARD*, conventional DMARD

^a^Thirty-seven people with RA provided 40 synovia including 3 individuals providing 2 separate synovia at different times from 13 to 16 months apart

^b^Percentage values for rheumatoid factor (RF) or anti-citrullinated peptide (CCP)^+^ are from 21/37 patients (57%) and 19/37 (51%) providing synovia

^c^Of patients providing synovia, data for erosions are from 24/37 patients (65%), and data for nodules are from 25/37 patients (68%)

^d^Disease-modifying anti-rheumatic drugs (DMARD) reconciled at the time of tissue collection were available from 25/37 of patients (68%)

### Tissue collection and processing

Freshly excised synovial membrane tissues were prepared for biobank, histology or gene expression workflow, as previously described [32].

### Gene expression analysis—real-time quantitative reverse transcription PCR (RT-qPCR) and droplet digital PCR (ddPCR)

RNA was extracted with on-column RNase-free DNase digestion and reverse transcribed as previously described [33]. Primers designed for specific termini of known *TP53* mRNA (*FL/Δ40TP53_T1*, *FL/Δ40TP53_T2*, *TP53α*, *TP53β* and *Δ133TP53*) were used, as previously documented [34]. RT-qPCR was performed using the LightCycler 480 System (Roche Diagnostics, USA) with SYBRGreen Master Mix (TaKaRa Bio). Relative expression levels were quantified by the ΔC_T_ method, with normalisation to endogenous control genes *GAPDH*, *HPRT1* and *ACTB* [26]*.* Absolute mRNA abundance of *Δ133TP53*, *TP53β* and *GAPDH* was measured using ddPCR with EvaGreen SuperMix on a Bio-Rad QX200 ddPCR System (Bio-Rad, USA). Target mRNA expression was converted to copies/μg RNA and normalised to *GAPDH* as previously described [34, 35].

### Plasma collection and processing for cytokine measurement and analyses

#### Human

Levels of Th17-related cytokines were measured in EDTA-plasma samples obtained prior to joint replacement surgery using a 15-plex magnetic bead-based Bio-Plex Pro Human Th17 Cytokine Panel (171AA001M), on a BioPlex 200 system (Bio-Rad). Complete data analysis was performed using the MilliplexAnalyst software (VigeneTech Inc., USA).

#### Mouse

Cytokine measurement, data analysis and validation of data reduction settings were carried out as previously described [32]. The sera from 9-week-old Δ122p53/ + (*n* = 4) and p53 + / − (*n* = 4) mice showing no obvious pathology [32] were analysed for the expression of cytokines and chemokines using a Bio-Plex Pro Mouse Cytokine 23-plex Assay #M60009RDPD (Bio-Rad).

### Western blot

Total protein lysates from PC-3 cells transiently transfected with either empty vector (Vo), or encoding Δ133p53α, β or γ, as previously described [26] were separated on Bolt 4–12% Bis–Tris Plus Gels (Invitrogen) and transferred onto nitrocellulose membranes, blocked with Odyssey Blocking Buffer, incubated overnight in primary antibody (1:1000) and detected using the secondary antibody IRDye 800CW goat anti-rabbit IgG (LI-COR Biosciences, USA). The membranes were analysed using the Image Studio software (LI-COR Biosciences, USA). The specificity of rabbit p53β (79.3) and Δ133p53 (KJCA133αβγ) antibodies (courtesy of Bourdon Laboratory, Jacqui Wood Cancer Centre, University of Dundee, UK) was established by Western blot analysis (Additional file 1: Fig. S3).

### Immunohistochemical (IHC) staining of RA synovial tissue

Formalin-fixed paraffin-embedded (FFPE) RA synovial tissue sections were stained with haematoxylin and eosin (H&E). For IHC, sections were incubated with antibodies specific for B cells (CD20, [Dako L26]; 1:20), T cells (CD3 [Cell Marque MRQ-39]; 1:50), macrophages (CD68 (Leica KP1); 1:100) and endothelial cells (vWF (Dako A0082); 1:600) diluted in Primary Antibody Diluent BOND (Leica Biosystems), using automated IHC following heat-mediated epitope retrieval and diaminobenzidine chromogen (DAB; Leica) or Bond Polymer Refine Red (Leica Biosystems) detection. For p53β, IHC was done manually following antigen retrieval (heat-mediated Tris–EDTA, pH 9.0) using rabbit polyclonal antibody ‘79.3’ (1:150) in van Gogh diluent (Biocare Medical) overnight at 4 °C, detection by EnVision Dual Link (Dako) followed by DAB (Dako) with DAB enhancer (Leica Biosystems).

### Immune cell infiltration and synovial tissue classification

Semi-quantitative assessment of immune cell infiltration was performed on RA synovial membrane IHC-stained tissue sections by grading positively labelled immune cells, as described in Additional file 1: Fig. S2, thus generating an immunoscore for each sample by adding the score from each B cell, T cell and macrophage category. B cells (CD20^+^) were graded in 3 categories: (i) number of follicles, (ii) number of clusters (a group of 5–15 cells) and (iii) number of scattered/diffuse cells (as % of tissue area). T cells (CD3^+^) were graded in the categories ii and iii above. Macrophages (CD68^+^) were graded based on the numbers of CD68^+^ cells within the synovial lining layer (MLS) together with the numbers of CD68^+^ single cells.

### Immunofluorescence staining

For cell types in RA synovia expressing p53β, sequential double-labelling was performed. Sections were permeabilised following antigen retrieval (Tris–EDTA pH 9.0) using 0.5% TritonX-100/1% BSA prior to blocking in 5% normal goat serum and incubated in primary antibody specific for p53β (79.3, 1:200 in van Gogh diluent). Following labelling with AlexaFluor 488 secondary antibody (1:1000; Life Technologies, USA) and a second serum blocking step, tissues were incubated with antibodies against CD55 (Abcam—EPR6689; 1:200), CD68 (Cell Marque-KP1; 1:500), CD90 (Abcam EPR3133; 1:200) or CD138 (Abcam—B-A38; 1:200). AlexaFluor 546 secondary antibody (Life Technologies, USA) was used to detect cell surface proteins. The nuclei were labelled with Hoechst dye (33,258; Thermo Fisher, USA).

Triple labelling was used to detect Δ133p53αβγ isoforms [36]. Sections were incubated with rabbit polyclonal antibody KJCA133p53 (1:300 in van Gogh diluent; Biocare Medical) followed by AlexaFluor 546 prior to incubation with 79.3 (p53β) and subsequent detection with AlexaFluor 488 before labelling of cell surface marker antibodies (CD55, CD68, CD138 or CD90) with the appropriate AlexaFluor 633. Specific signal in both 546 nm and 488 nm channels indicated Δ133p53αβγ isoform detection, while independent signal in 633 nm indicated cell type.

### Quantification of Δ133p53β expressing cells

Labelled RA synovial sections were imaged and quantitated using the Lionheart FX Automated Live Cell Imager (BioTek, Winooski, VT, USA) equipped with a 20 × 0.45 NA air objective. Cells stained for p53β (488 nm) and either CD55, CD68, CD90 or CD138 were analysed for fluorescence intensity (546 nm) using the dual masking capability of the Gen5 3.0 software. Cells labelled with Δ133p53β and either CD55, CD68, CD90 or CD138 were imaged on a Zeiss 710 confocal laser scanning microscope. Ten images per combination were taken at × 20 magnification, with at least 10 cells expressing p53β per image, ensuring an average of at least 100 cells counted for each staining combination. Quantitation of cells labelled with a combination of Δ133p53 (546 nm) and anti-p53β (488 nm) and either CD55, CD68, CD90 or CD138 (633 nm) was done in ImageJ.

### RNAScope in situ hybridisation for *Δ133TP53β* mRNA expression

A custom-made probe specific for the unique region of *Δ133TP53* and *TP53β* was designed to the *Δ133TP53β* reference sequence DQ186651.1 [27] (Advanced Cell Diagnostics (ACD); USA). Serial 5-μm sections were probed with the target, a negative control probe *DapB* (31,143, ACD) and positive control *Ubiquitin C* (Hs-*UBC*; 310,041, ACD). RNAscope in situ hybridisation (ISH) was performed according to the manufacturer’s instructions utilising RNAscope 2.5 HD Reagent kit-Brown (ACD) (Leica Biosystems, Germany). Sections were scanned using the Aperio ScanScope CS digital pathology system (Leica Biosystems, Germany)**.**

### Statistical analysis

A complete linkage hierarchical clustering was performed using the hclust2() in R (R Core Team, 2017), and figures were produced using the package ggplot2, after ranking mRNA expression of *FL/Δ40TP53_T1*, *FL/Δ40TP53_T2*, *Δ133TP53*, *TP53α* and *TP53β* in ascending order using the rank(). One-way ANOVA for multiple comparison analysis and display of double and triple IF staining was performed in GraphPad Prism (version 7.03, GraphPad Software, San Diego, USA). *TP53* isoform mRNA expression or cytokine/chemokine levels were determined using unpaired *t* test with Welch’s correction with GraphPad Prism. Spearman correlation analysis to explore associations between gene expression levels and plasma cytokine levels were performed in GraphPad Prism.

## Results

### *Δ133TP53* and *TP53β* mRNA levels are highest in synovial tissue with extensive inflammation

We compared *TP53* isoform expression in RA synovia with that in OA and found levels of *TP53* isoform mRNA vary, with ~ 100-fold more expression of the *TP53β* and *FL/Δ40TP53_T2* mRNAs than of *FL/Δ40TP53_T1*, *Δ133TP53* and *TP53β* mRNAs (Additional file 1: Fig. S4). Levels of *Δ133TP53* and *TP53β* mRNA are significantly greater in RA synovia when compared to OA, suggesting higher expression of these isoforms is a feature of RA.

**Fig. 1:**
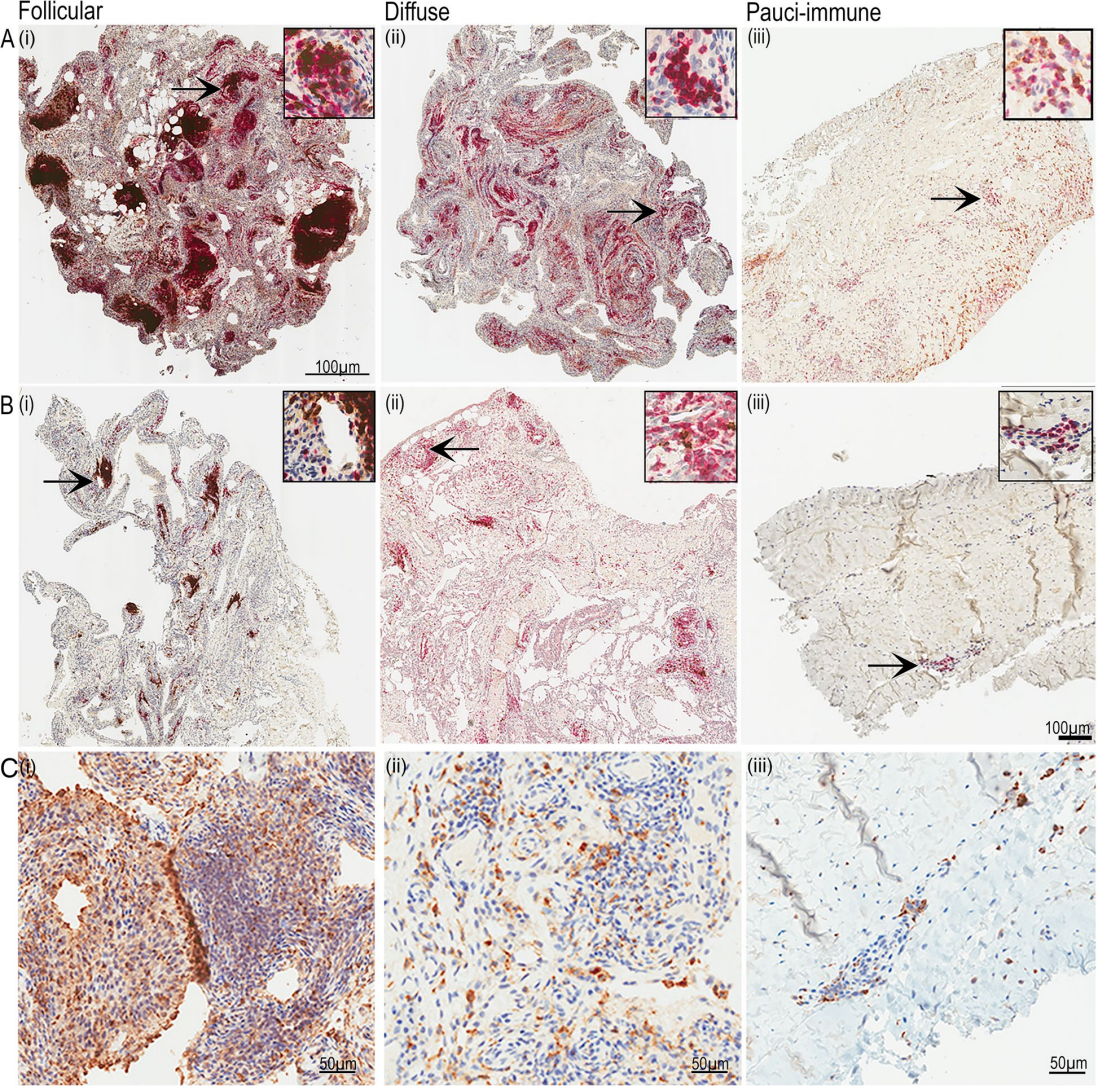
RA cohort divided into three synovial membrane histopathological subtypes which display different immune phenotypes, based on immune cell infiltrate. B cells and T cells were identified in duplex IHC by antibodies to CD20 (brown) and CD3 (red) (**A**, **B**). Macrophages/MLS identified by CD68 (brown) (**C**). Follicular: **A** (i) with many ectopic lymphoid structures (ELS) and T and B cell aggregates or **B** (i) small number of ELS and T- and B cells. Diffuse: **A** (ii) dominated by myeloid cells, T- and B-lymphocyte aggregates and scattered T and B cells; **B** (ii) small numbers of T- and B cell aggregates and scattered T- and B cells. Pauci-immune: **A** (iii) fibroblast dominated, with minimal inflammatory infiltrate areas of scattered T- and B cells; **B** (iii) very occasional T- and B cells. **C** CD68 + cells in (i) follicular, (ii) diffuse and (iii) pauci-immune subtypes. Arrows indicate the location of the inset (enlarged) images

Subsequently, we determined whether the level of *Δ133TP53* and *TP53β* mRNA expressed in RA synovia associates with particular histopathological sub-types of synovial inflammatory cells. The presence of infiltrating CD20^+^ B cells, CD3^+^ T cells and CD68^+^ macrophages was determined using IHC. Rheumatoid synovial tissues were further classified into one of three pre-defined subtypes based on immuno-histopathology: (i) follicular—predominantly lymphoid with ectopic lymphoid structures (ELS); (ii) diffuse—dominated by myeloid cells and T lymphocytes but with more limited B lymphocyte involvement; or (iii) pauci-immune—fibroblast dominated with minimal inflammatory infiltrate [37]. Representative images of the three histopathological subtypes are shown in Fig. 1. Based on our assessment criteria, from 33 FFPE RA synovia, a total of 12 (36%) synovia were classified as follicular, 8 (24%) were diffuse and 13 (39%) were pauci-immune (see Additional file 1: Fig. S2).

**Fig. 2:**
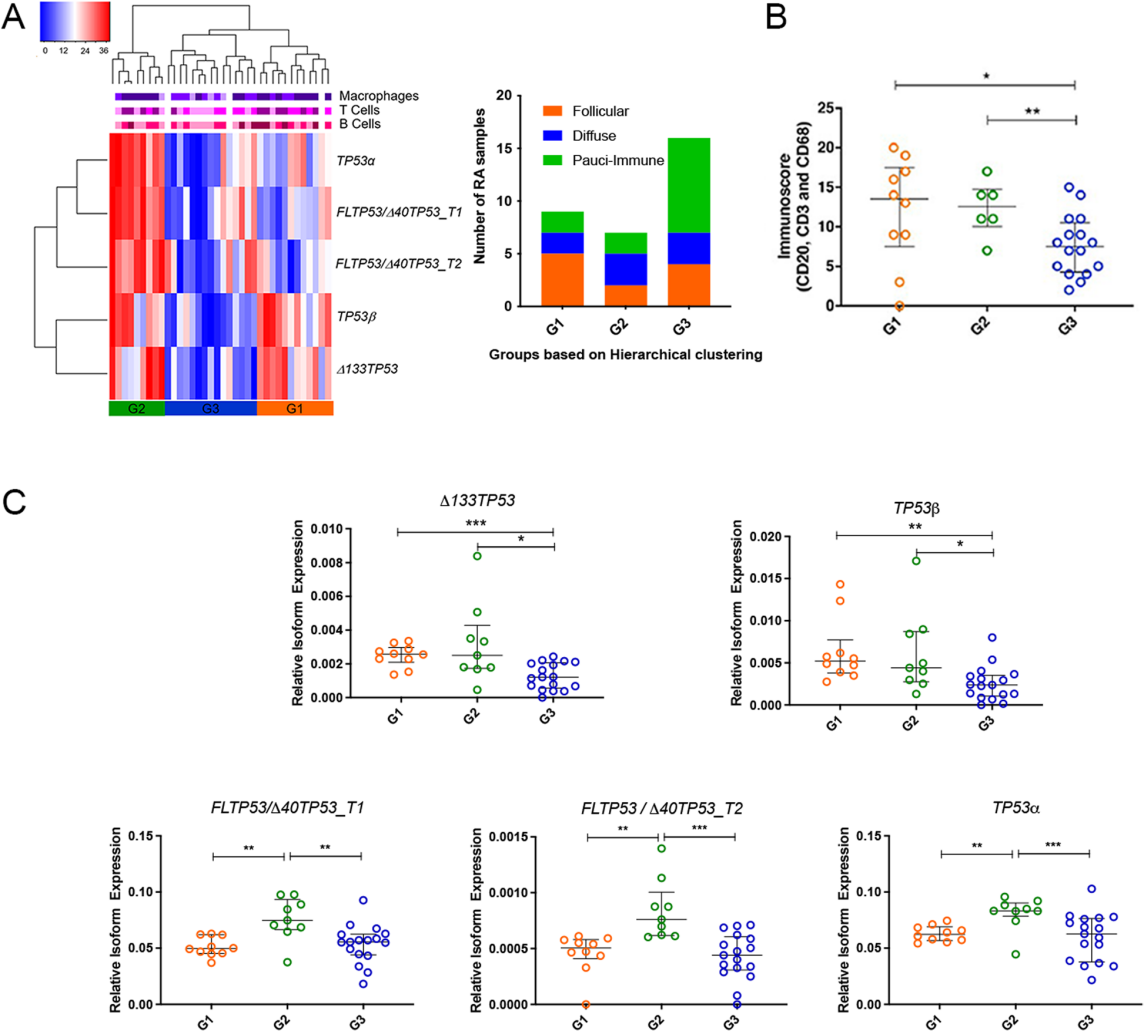
RA samples with elevated *Δ133TP53* and *TP53*β mRNA expression are associated with high immune infiltration. **A** Hierarchical clustering analysis of RA joint tissue by mRNA expression of *FL/Δ40TP53_T1*, *FL/Δ40TP53_T2*, *Δ133TP53*, *TP53α* and *TP53β*. The coloured label bars on the top indicate the amount of infiltration of CD68^+^ cells, CD3^+^ T cells and CD20.^+^ B cells (warm colours: high infiltration; cool colours: low infiltration). The bar graph represents the number of RA patient samples from each of the RA subtypes that fall within the groups G1, G2 or G3, determined by rank hierarchical clustering. **B** Immunoscore vs transcript expression for each patient sample. **C** Transcript expression of *Δ133TP53*, *TP53β*, *FL/Δ40TP53_T1*, *FL/Δ40TP53_T2* and *TP53α.* Dots in **B** and **C** represent the individual data points in each group. The lines represent the mean and SD. Significance was determined using an unpaired *t* test with Welch’s correction. *p < 0.05; **p < 0.01

To explore the potential associations between expression levels of various *TP53* isoform mRNA and the presence of infiltrating CD20^+^ B cells, CD3^+^ T cells and CD68^+^ macrophages, we carried out rank ordered hierarchical clustering on normalised *TP53* isoform expression from 36 RA samples, of which we had immune cell infiltration data for 33. This analysis identified three main clusters, nominally designated G1, G2 and G3 (Fig. 2A), which varied in the extent of immune cell infiltration (Fig. 2B) and individual *TP53* isoform expression (Fig. 2B, C). The bar graph (Fig. 2A), shows the number of RA patient samples in each of these clusters, based on the RA subtype. The extent of immune cell infiltration in each of the clusters was determined using an immunoscore based on IHC staining of overall infiltration by T and B cells and macrophages. Amongst samples in G1 and G2 clusters, on average, significantly higher immunoscores (Fig. 2B), and significantly higher levels of mRNA *Δ133TP53* and *TP53β* isoform mRNA (Fig. 2C), were evident when compared to synovia clustering as G3. However, the levels of *FL/Δ40TP53_T1*, *FL/Δ40TP53_T*2 and TP53α mRNA were similar between the G1 and G3 clusters (Fig. 2C). In contrast, there is significantly higher mean expression of *FL/Δ40TP53_T1*, *FL/Δ40TP53_T2* and *TP53α* mRNA in the synovia grouped in the G2 cluster (Fig. 2C) compared to G1 or G3 clusters. These data suggest that higher levels of *Δ133TP53* and *TP53*β mRNA are more likely to be associated with the follicular and diffuse subtypes, alongside an increase in the immunoscore.

### *Δ133TP53β* mRNA and p53β protein are expressed in multiple cell types involved in RA

**Fig. 3:**
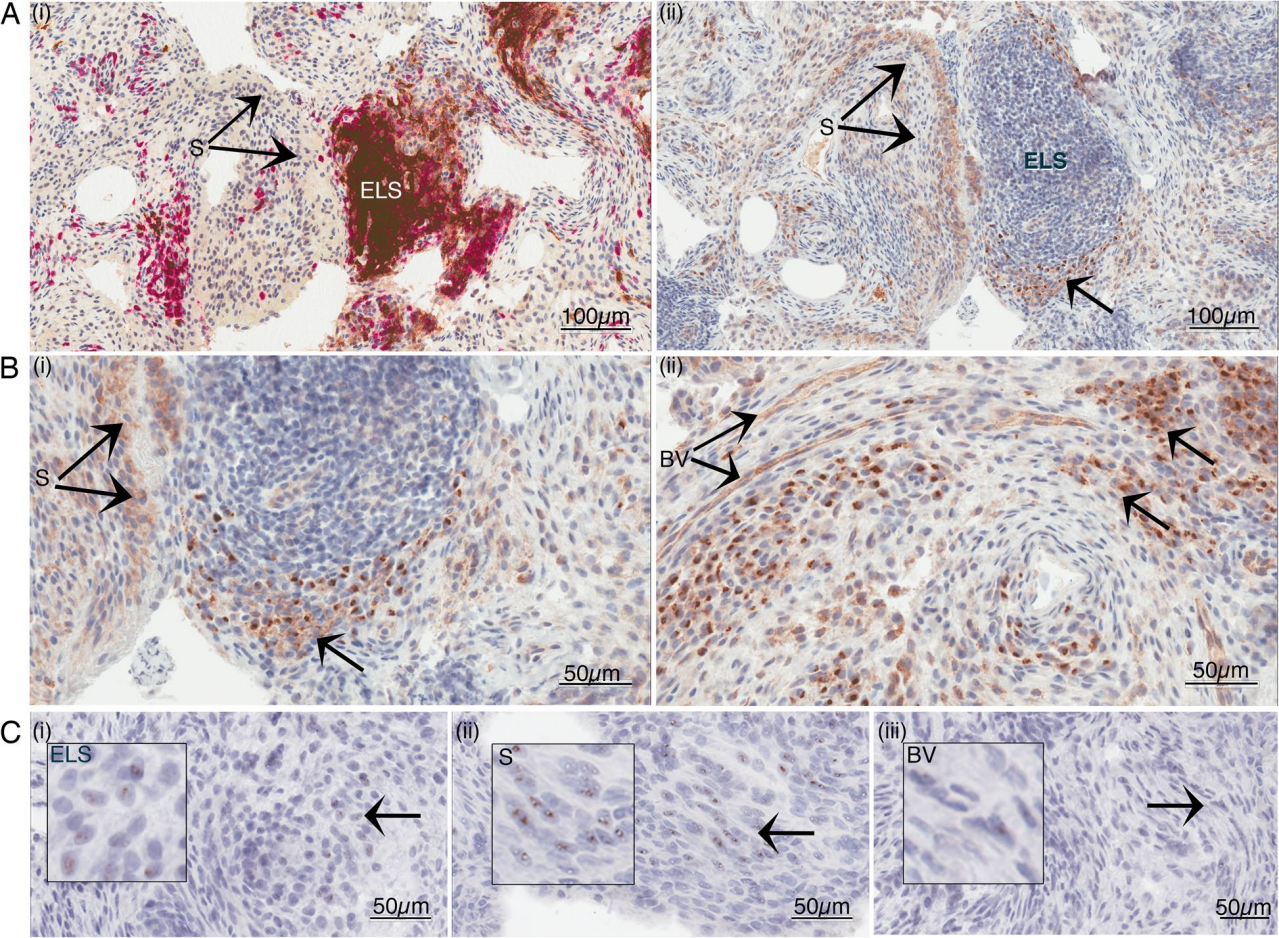
Δ133p53β gene expression seen in cells and areas positive for p53β protein. **A** IHC staining for (i) CD20^+^ B cells (brown) and CD3^+^ T cells (red) and (ii)79.3^+^ p53β protein expression; × 200 magnification. **B** IHC p53 higher magnification with (i) synovial cells and (ii) blood vessels displaying moderate granular staining and intense polarised staining seen in individual cells surrounding ELS, below the blood vessels and below the synovium as indicated; × 400 magnification. **C** RNAScope *Δ133TP53* message evident (i) within and around ELS and endothelial cells of the blood vessels and (ii) within the synovial lining cells and in the synovial sub-lining cells; × 400 magnification. S, synovial lining; SS, synovial sub-lining; ELS, ectopic lymphoid structure; BV, blood vessel. Arrow represents the area immediately below the synovium

To identify cells expressing Δ133p53β protein within RA synovial tissues, IHC was first carried out using an antibody that specifically detects all p53β protein variants (Additional file 1: Fig. S5) [36]. As expected, quintessential histological features accompany the increased immune cell infiltration in follicular synovial subtypes, including the presence of ELS (Figs. 1 and 3A(i)), and distinct proliferative synovium (Fig. 3A (ii) and B (i) higher magnification). In these synovia, the p53β protein is localised to the endothelium (blood vessels displaying moderate granular staining) and is also apparent as intense staining within individual cells surrounding ELS (Fig. 3A (ii) and at higher magnification 3B (i)) and elsewhere within the synovial sub-lining (as shown by arrows in Fig. 3B (ii). RNAScope-ISH analysis, used to identify *Δ133TP53* mRNA, shows a similar pattern, with specific transcript evident in endothelial cells and in cells positioned within and near ELS (Fig. 3C (i)) and in the synovial sub-lining regions (Fig. 3C (ii)). Conversely, *Δ133TP53* mRNA was less frequently observed in the synovial lining regions (Fig. 3C (ii)). The combined analyses suggest the presence of *Δ133p53* and p53β isoforms is associated with similar cell types and with areas of more densely infiltrating and organised inflammatory infiltrate involving B and T cells.

### CD90+ activated fibroblasts specifically express *Δ133p53β* isoform and show unique cellular localisation

**Fig. 4:**
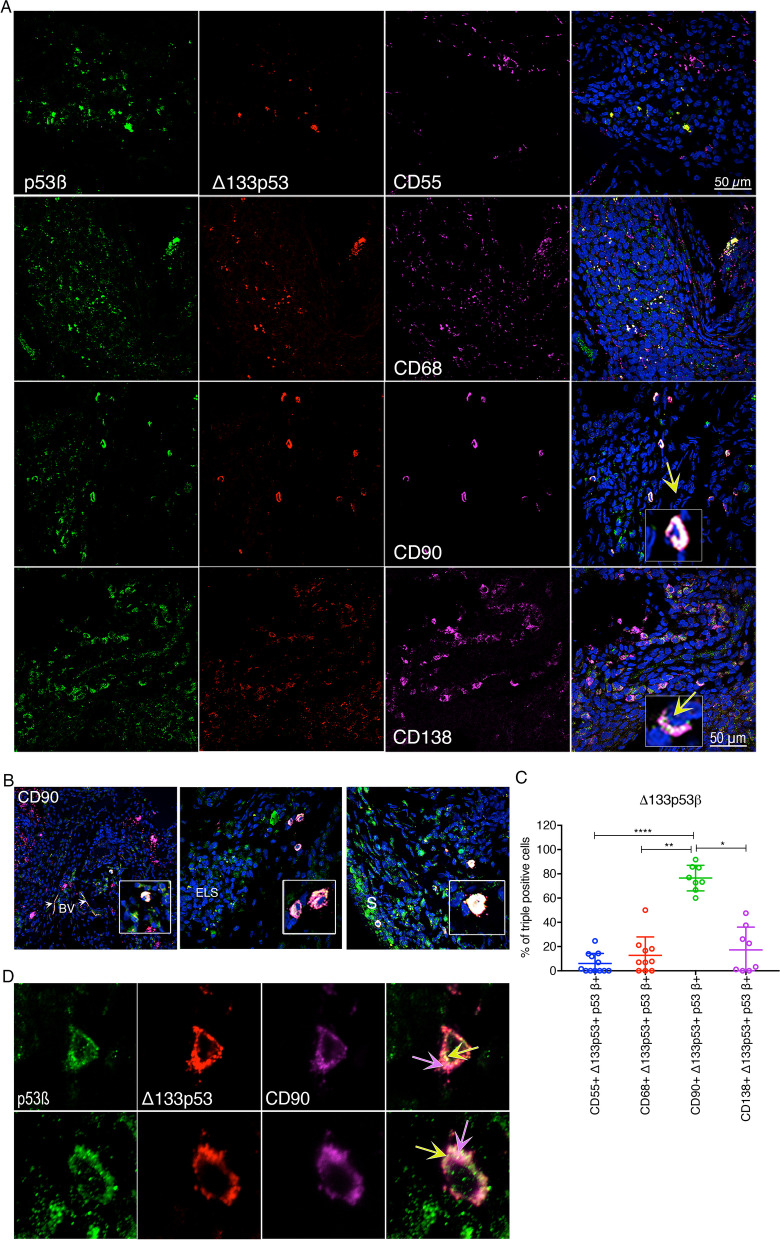
CD90^+^ FLS express the highest levels of Δ133p53β isoform. **A** p53β expressing cells (green, panel 1), Δ133p53αβγ expressing cells (red, panel 2), cells expressing either CD55^+^, CD68^+^ and CD90^+^ or CD138^+^ (magenta, panel 3); merged images (panel 4) showing co-localisation of p53β with Δ133p53β (yellow) and CD90^+^/Δ133p53β^+^ co-localisation (light pink); × 400 magnification, scale bar 50 μm. **B** Regional localisation of CD90^+^/Δ133p53β^+^ cells (light pink) adjacent to the blood vessels (BV, left panel), ELS (middle panel) and synovium and sub-lining (S, right panel); × 400 magnification, scale bar 50 μm. **C** The percentage of CD55^+^, CD68^+^, CD90^+^ and CD138^+^ cells, represented in **A**, which co-express p53β, Δ133p53 and both Δ133p53 and p53β. Each dot represents the results from each individual image field and a minimum of 100 cells. Lines represent the mean and standard deviation. Significance was determined using Kruskal–Wallis with Dunn’s multiple comparison (*p < 0.002, **p < 0.005 and ****p < 0.0001). **D** Δ133p53β isoform is located in CD90^+^ FLS. Images from the top and bottom rows show the cellular location of p53β (green, panel 1), Δ133p53αβγ (red, panel 2) and CD90 (magenta, panel 3). Panel 4 represents the merge of panels 1, 2 and 3, demonstrating the location of Δ133p53αβγ and p53β in CD90^+^ cells. Co-localisation of Δ133p53αβγ with p53β is yellow (yellow arrow) and represents Δ133p53β; note the large perinuclear aggregates; co-localisation of Δ133p53β (yellow) CD90 (magenta) is light pink (light pink arrow); co-localisation of Δ133p53αγ with CD90 is shown as a deeper pink. Panels 1 through 4 indicate panel position from left to right

Having determined that both *Δ133TP53* mRNA and p53β protein are found in similar regions and cells in RA synovia, further analysis was carried out to establish if the specific staining detected was that of the Δ133p53β protein isoform. Various cell types within the RA synovium can be distinguished based on the expression of cell surface markers. CD55 is a cell membrane-bound protein expressed on subsets of fibroblasts (FLS) that line the synovial membrane. CD90 is expressed on a distinct subset of fibroblasts confined to the perivascular regions of the synovial sub-lining area and around ELS. Some studies have shown CD90^+^ fibroblasts can exhibit a tumour-like behaviour [31, 38, 39]. and their presence and function have been associated with lymphoid pathology [31, 40–42]. CD68 is a pan macrophage marker, which we used to identify macrophages and macrophage-like synoviocytes (MLS), while CD138 identifies plasma cells, also shown to associate with lymphoid pathology and auto-antibody production in RA [43]. To identify and characterise cell types expressing the Δ133p53β protein isoform, we performed triple immunofluorescence (IF) labelling to distinguish CD55^+^ lining fibroblasts, CD90^+^ fibroblasts, CD68^+^ macrophage subpopulations and CD138^+^ plasma cells. Antibodies to each of these cell markers were used in combination with a rabbit polyclonal antibody (KJCA133αβγ) binding to Δ133p53αβγ and with a second polyclonal rabbit antibody (79.3) against p53β (Fig. 4) [36].

**Fig. 5:**
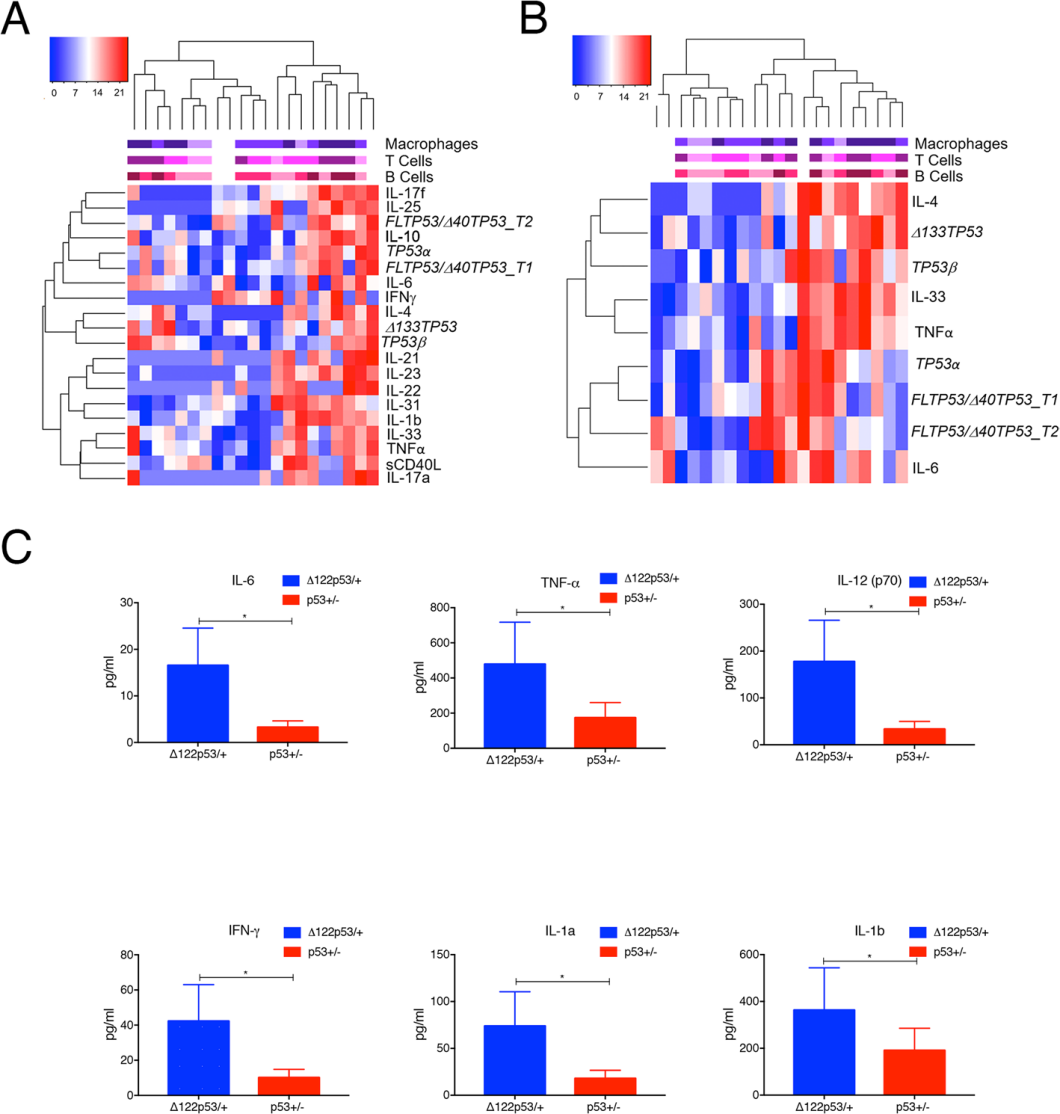
RA synovial tissue with elevated *Δ133TP53* and *TP53β* transcript expression is associated with elevated levels of plasma cytokines. **A**, **B** Hierarchical clustering analysis of RA synovial tissue by mRNA expression of *FL/Δ40TP53_T1*, *FL/Δ40TP53_T2*, *Δ133TP53*, *TP53α* and *TP53β.* **A** Fifteen T_h_17-related plasma cytokines. **B** Four selected plasma cytokines. The scale labels along the top indicate the amount of infiltration of CD68^+^ cells, CD3^+^ T cells and CD20^+^ B cells (warm colours: high infiltration; cool colours: low infiltration). **C** Levels of 6 serum cytokines (IL-6, TNF-α, IL-12(p70), IFN-γ, IL-1a and IL-1b) from 9-week-old Δ122p53 + / − (blue) and p53 + / − (red) mice. Shown are the mean concentrations of each cytokine from serum pooled from 4 individual mice with error bars at 97% CI. Significance was determined using an unpaired *t* test with Welch’s correction; *p < 0.05

As expected, CD55^+^ fibroblasts were found predominantly in the synovial lining of the tissue, and CD68^+^ macrophages were found both in the synovial lining and scattered throughout the tissue (Fig. 4A, rows 1–2; Additional file 1: Fig. S5 A-B, Rows 1–2). CD90^+^ fibroblasts were found in the sub-lining areas of hyper-proliferative synovium, particularly in the perivascular areas, as well as surrounding the ELS (Fig. 4A, row 3, and Fig. 4B; Additional file 1: Fig. S5C, Rows 1–2). Endothelial cells co-expressing von Willebrand factor (vWF) and CD90^+^ were evident (Additional file 1: Fig. S5C, Row 3), and typically, endothelial cells also demonstrated p53β expression (Additional file 1: Fig. S5C, Row 2). Plasma cells (CD138^+^) were observed towards the outer regions of ELS and near blood vessels (Fig. 5A, row 4).

Comparison of the frequency of p53β detection within and between cell types showed the cell type with the highest proportion of cells co-expressing p53β were the CD90^+^ sub-lining fibroblasts (76 ± 10%; mean ± SD), followed by CD68^+^ macrophages and MLS (41 ± 12%) and then CD55^+^ lining fibroblasts (18 ± 9%; Additional file 1: Fig. S5D). Statistically significant differences in the frequency of p53β co-expression were observed between all cell types.

To identify the proportion of cells that co-expressed p53β and Δ133p53(αβγ) isoforms, we quantitated the triple IF labelling in CD55^+^, CD68^+^, CD90^+^ and CD138^+^ cells (Fig. 4A, B). Our analysis showed that the cell type most frequently expressing the Δ133p53β protein isoform was the CD90^+^ sub-lining fibroblasts (77 ± 11%), followed by CD138^+^ plasma cells (19 ± 18%), CD68^+^ macrophages (15 ± 13%) and CD55^+^ fibroblasts (8 ± 6%; Fig. 4C). These combined analyses suggest that CD90^+^ fibroblasts appear to preferentially express the Δ133p53β isoform, compared to CD55^+^ fibroblasts, CD68^+^ macrophages and CD138^+^ plasma cells (Fig. 4C). Of note, individual CD90^+^ cells co-expressing the Δ133p53β isoform were present within the synovial sub-lining and near perivascular areas and surrounding ELS (Fig. 4B, left to right respectively).

### CD90^+^ cells express *Δ133p53β* as large peri-nuclear aggregates

To determine the intracellular location of CD90 in relation to Δ133p53β, we performed maximal and 3D projections of confocal images of tissue sections triple-stained with antibodies to CD90/Δ133p53αβγ/p53β (Fig. 4D). During this analysis, we found examples of cells displaying triple-positive peri-nuclear aggregates inferred to be comprised of CD90 and the Δ133p53β protein isoform (Fig. 4D). In addition, these CD90^+^ cells also express p53β alone, or other Δ133p53 isoforms, including α and or γ (Fig. 4D).

### RA synovia with elevated *Δ133TP53* and *TP53β* expression are associated with elevated levels of plasma cytokines

Elevated levels of circulating proinflammatory cytokines are a recognised feature of RA. Consequently, we measured the plasma levels of fifteen T_h_17-related cytokines from 21 participants within our RA cohort. Using unsupervised hierarchical clustering, we assessed whether the cytokine levels were associated with synovial tissue expression levels of *Δ133TP53* and *TP53β* isoform mRNA and immune cell infiltration. Clustering analysis distinguished two main groups; one group which had higher levels of mRNA transcript for *TP53* isoforms in synovial tissue and concurrently high levels of certain plasma cytokines (Fig. 5A). Subsequent Spearman correlation analyses further distinguished the plasma cytokines TNFα, IL-4, IL-6 and IL-33 to show statistically significant positive correlations with synovial *Δ133TP53* or *TP53β* mRNA expression (Additional file 2: Table S1). Plasma TNFα levels positively correlated exclusively with synovial *Δ133TP53* mRNA expression, while plasma IL-6 positively correlated with both synovial *TP53α* and *TP53β* isoform expression. Other plasma cytokines, including IL-1β, IL-10, IL-23 and IL-17F varyingly correlated with the synovial expression of *FL/Δ40TP53_T1* and *FL/Δ40TP53_T2* or *TP53α* isoforms (Additional file 2: Table S1). A further, more refined, hierarchical clustering analysis was also performed, using only those plasma cytokines displaying significant associations with *Δ133TP53* or *TP53β* synovial mRNA expression (Fig. 5B). Consistent with the correlation analysis, the RA tissues were divided into two groups, where one group had elevated levels of *Δ133TP53* or *TP53β* synovial mRNA expression and increased levels of TNFα, IL-4, IL-6, and IL-33, compared to the other group.

These data are complemented by further analysis and comparisons of serum cytokines in the mouse Δ122p53 model of Δ133p53 isoforms, which displays extensive inflammatory pathologies [29]. Comparison between heterozygous Δ122p53 (Δ122p53/ +) mice and heterozygous wild type (p53 + / −) mice shows that over time, the Δ122p53/ + mice exhibit spontaneous increases in multiple circulating cytokines and chemokines (Fig. 5C and Additional file 1: Fig. S6), including pro-inflammatory cytokines IL-6, TNFα, IL-12p70, IFNγ, IL-1α and IL-1β (Fig. 5C). Combined, these results suggest that synovial *Δ133TP53* and *TP53β* isoform expression is associated with a greater extent of systemic inflammation in RA, including the involvement of the key pro-inflammatory mediators, TNFα and IL-6.

Amongst genes analysed within synovial tissue, only *IL27B/EBI3* transcript levels significantly correlated with *Δ133TP53* and *TP53β* isoform mRNA expression (Additional file 2: Table S1). Excess IL27B/EBI3 is a trait of plasma cells [32] that feature within highly inflamed synovia and is associated with ELS formation and increased disease severity [44]. Also notable, the expression of *NOTCH3* and *JAG1*, reflecting synovial NOTCH activation critical to the differentiation of CD90^+^ synovial fibroblasts [45], showed no correlation with *Δ133TP53* or *TP53β* expression. Our results are consistent with the activation of circulating cells prior to their infiltration of involved joint synovial tissue, including the possibility that pre-inflammatory mesenchymal (PRIME) cells are involved [46].

## Discussion

Our initial analysis comparing OA and RA synovial tissues established greater expression of the Δ*133TP53* and *TP53β* mRNAs associated with rheumatoid synovial inflammation. Moreover, we found that elevated levels Δ*133TP53* and *TP53β* mRNAs were associated with increased immune cell infiltration more frequently observed in follicular and diffuse subtypes of RA. Further analysis using RNAscope-ISH to detect the unique regions of *Δ133TP53* and *TP53β* mRNA transcripts showed expression in particular cell types including endothelial cells, but otherwise confined to specific locations within the inflamed rheumatoid synovium, most obviously the perivascular areas of the synovial sub-lining, and around and within ELS. A similar staining pattern was evident using IHC with antibodies that specifically detect Δ133p53 or p53β protein isoforms. We identified CD90^+^ fibroblasts as the cell type most commonly expressing both the Δ133p53 and p53β protein isoforms (~ 76%) in RA synovium. This suggests the predominant isoform expressed by these cells is Δ133p53β [23, 25, 26]. We find that the CD90^+^ cells, co-expressing both Δ133p53 and p53β, are located immediately adjacent to the CD90^+^ blood vessels and predominantly within the perivascular areas of the synovial sub-lining and surrounding the ELS. p53β isoforms were found in aggregates which may be important in regulating p53β function [47]. An earlier study described an almost identical localisation for CD90^+^ cells within the RA synovium [31] where they were also shown to play a key ‘pathogenic’ role in the course and severity of RA. Our data suggest an association between the CD90^+^ fibroblasts expressing the Δ133p53β isoform and high inflammatory activity in RA, reflected systemically by the levels of circulating proinflammatory cytokines, including TNFα and IL-6 and locally by greater immune cell infiltration of involved joint synovial tissue. Heightened systemic inflammation is also present in the murine Δ122p53 model of Δ133p53 isoforms where our data indicate there is an impact on a diverse array of inflammatory mediators [29].

Systemic B cell activation is one consequence of heightened inflammation in RA and has been linked to the presence of circulating CD45^−^ CD31^−^ PDPN^+^ pre-inflammatory mesenchymal (PRIME) cells. The PRIME cells share features of CD90^+^ inflammatory synovial fibroblasts and are potentially the precursors of such cells [46]. Typically, the heightened immune cell infiltrate associated with synovial Δ133p53β expression includes B cells and is well organised, generally featuring prominent ELS that define a synovial follicular subtype. Previously, we have linked the co-expression of *CD21L* and *IL17A* to the number and size of B cell clusters in synovial tissue and high inflammation [43]. However, we find no links between *CD21L* or *IL17A* expression in synovial tissue and any of the *TP53* isoforms. Similarly, we found no association between synovial *Δ133TP53* or *TP53β* mRNA expression and localised expression of limited proinflammatory genes including *TNF-α*, *IL6* or *IL27A*. Significant correlations were evident between *IL27B/EBI3* (also known as EBV-induced gene 3; also known as *IL35B*) expression and the expression of both *Δ133TP53* and *TP53β* mRNAs. In RA synovium, the most obvious presence of IL27B/EBI3 protein is with plasma cells (PC) at the periphery of ELS [48] and likely acquired during PC differentiation [49]. Our data show that a subset of synovial PCs expresses the Δ133p53β isoform. Recent suggestions that synovial PCs may be recruited to the inflamed joint tissue [50] highlight the need for further investigation of the impact of Δ133p53 isoforms in systemic inflammation.

In RA synovium, up to 11 distinct and differentially abundant fibroblast clusters have been identified [50] with CD90 expression a key distinguishing feature in multiple studies. Thus, in human synovium, CD34 and CD90 expression distinguish CD34^−^ CD90^+^ fibroblasts [31], which behave similarly to human CD90^+^ HLA-DR^+^ fibroblasts [51] and to human and murine FAPα^+^ CD90^+^ fibroblasts [50, 52], in producing a unique repertoire of pro-inflammatory cytokines and/or chemokines that function to maintain synovial inflammation. The expression of the Δ133p53β isoform by CD90^+^ synovial fibroblasts is associated with this immune effector function adopted by CD90^+^ fibroblasts of sustaining inflammation. Limited studies have addressed the functional polarisation attributed to CD90^+^ fibroblasts. The transcriptome of CD90^+^ HLA-DR^+^ fibroblasts suggests a response to interferon signalling [51], while the FAPα^+^ CD90^+^ fibroblast subset (designated as ‘F11’) responds to the synergistic action of IL-1β and TGFβ [50]. While the latter reflects a potential interaction between infiltrating immune cells and CD90^+^ fibroblasts within the synovium, there is also evidence for crosstalk between synovial endothelial cells and CD90^+^ fibroblasts involving NOTCH activation [45]. Evidence from human cancers showed that those tumours with elevated expression of the Δ133p53β isoform were invariably associated with significant immune cell infiltration [26, 27]. In addition, the transgenic Δ122p53 mouse model of human Δ133p53 showed widespread inflammation, including lymphoid aggregates in multiple organs and elevated levels of pro-inflammatory serum cytokines, notably IL-6 [29, 30]. Notwithstanding that the mechanism initiating Δ133p53β isoform expression remains unclear, the combined data suggest that elevated Δ133p53β expression is a key activator of the pro-inflammatory features of the CD90^+^ fibroblasts and is clearly manifest in RA.

In astrocytes and in other cell types, a pro-inflammatory environment induces the expression of CD90 to generate a ‘reactive’ phenotype [53]. CD90 interacts with many ligands, plays a role in cell–cell and cell–matrix interactions, activation and apoptosis of T cells, leukocyte cell adhesion and migration, proliferation and migration of fibroblasts in wound healing, inflammation and fibrosis (reviewed in [54]). Increased CD90 expression is associated with other pathologies such as cancer [55–57], where it induces inflammation and increases tumour progression by promoting IL-6 secretion [58, 59]. CD90 is also expressed in ‘activated’ endothelium in a pro-inflammatory environment and promotes transmigration of cells such as leukocytes [60]. Thus, CD90^+^ fibroblasts are of central importance in promoting inflammation in different contexts, including RA [31, 60–62].

**Fig. 6:**
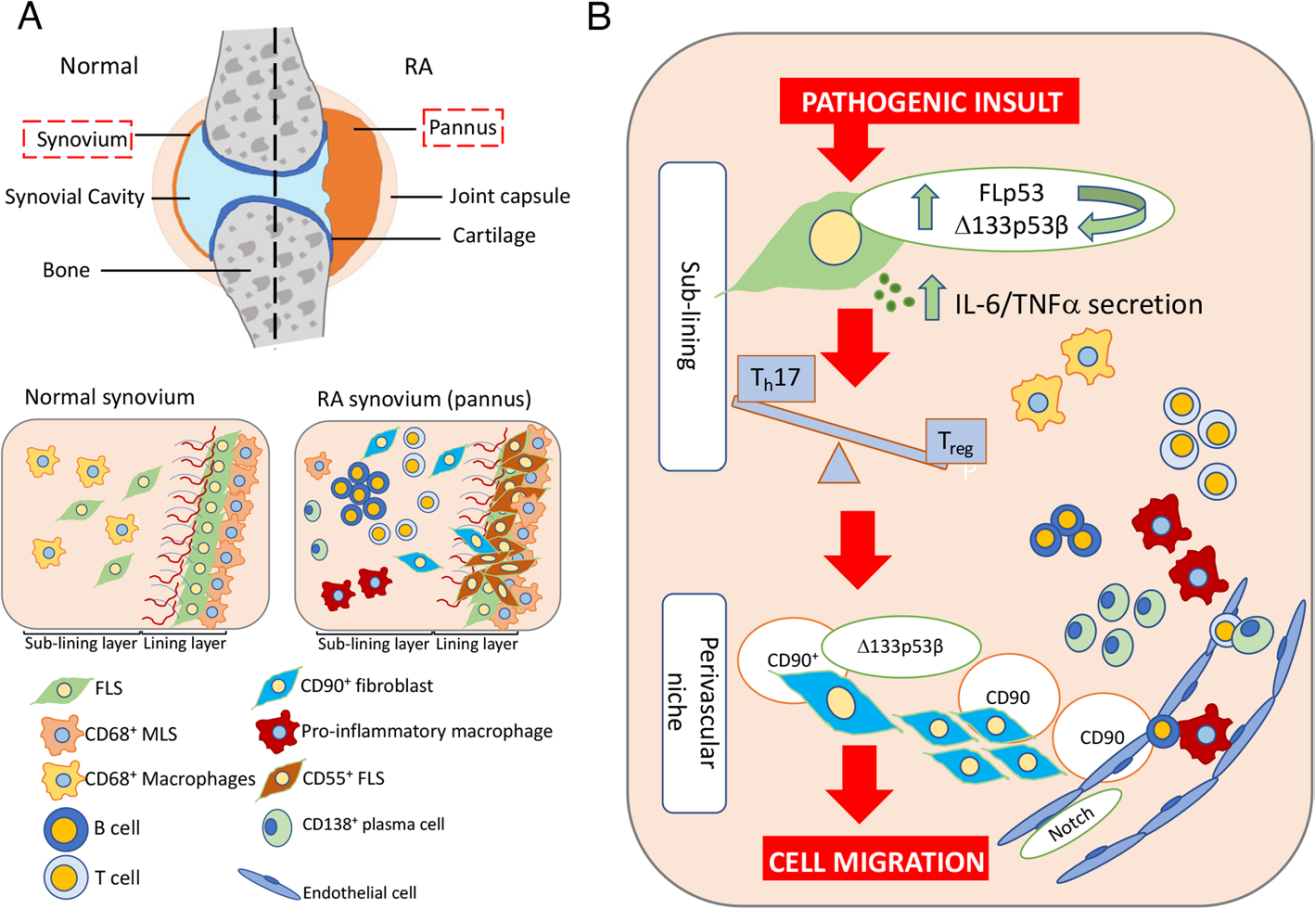
Model proposing how Δ133p53β orchestrates CD90 cell migration in RA. **A** Graphical representation of the normal and RA joint synovia. **B** The inflammatory milieu invokes a *TP53* response leading to an upregulation of Δ133p53β in FLS. This leads to cytokine secretion, including IL-6, sustaining a pro-inflammatory microenvironment. Downstream effects include the expansion and persistence of T_h_17 cells vs T_regs_ and the recruitment and activation of CD90^+^ FLS, resulting in their migration through the CD90^+^ perivasculature facilitated by CD90^+^ endothelial cells

We propose a model (Fig. 6) whereby the onset of the inflammatory process underpins disease progression in RA. Cells receiving a pathogenic insult (stress), invoke a p53-mediated response, increasing expression of Δ133p53β, which initiates an inflammatory response, including IL-6 production (tipping the balance in favour of T_h_17 cells) [63, 64] and in turn drives expression of CD90^+^ fibroblasts. Thus, elevated Δ133p53β expression is the key activator of the pro-inflammatory features of the CD90^+^ fibroblasts. This combination of factors in those more genetically susceptible maintains the stimulus, resulting in a persistent, prolonged inflammatory milieu that does not resolve. Such uncontrolled inflammation likely contributes to a more destructive RA phenotype. In this regard, the correlation between *Δ133TP53* or *TP53β* mRNA expression and serum IL-6 in people with RA suggests anti-IL-6 therapy may be the most appropriate targeted biological therapy in this group of individuals. Further clinical studies will be required to determine whether this is indeed the case and whether such targeted therapy alters synovial tissue *Δ133TP53* or *TP53β* mRNA expression.

## Conclusion

This study makes the novel observation that the RA synovial phenotype with the highest immune infiltrate, associated with a synovial follicular subtype and characterised by CD90^+^ migrating fibroblasts is, in part, caused by increased levels of the Δ133p53β isoform that drive inflammatory signalling pathways, including those regulated by IL-6. The study suggests that Δ133p53β expression could represent a biomarker for identifying people with RA who could benefit, for example, from anti-IL-6 treatments in the management of this disease.

## Supplementary Information

**Additional file 1:** **Fig. S1.** Schematic of *TP53* transcripts and protein isoforms. **A** Top panel shows the gene structure of *TP53*. The bottom panel shows the 9 *TP53* transcripts resulting from alternative splicing (α, β, and γ) and alternative promoter usage (P1 and P2). RT-qPCR regions are shown as a red arrow that correspond to the 5’ end of the *TP53* transcript for *FL/Δ40TP53_T1, FL/Δ40TP53_T2, Δ133TP53, *and those to the 3’ end for *TP53*α, *TP53*β and *TP53*γ. Light blue region represents the coding exons; grey regions represent the untranslated regions. **B** Region recognized by the rabbit polyclonal antibody KJCA133αβγ and 79.3 designed to specifically detect the Δ133p53 and *TP53*β isoform families respectively. **Fig.** **S2.** Classification of RA subtype based on IHC. Immunoscores were assigned based on the following criteria: (i) B cell (CD20): Follicles: [0: none evident]; [1: <5]; [2: 5-10]; [3: >10]. (ii) Clusters: [0: negative]; [1: low]; [2: medium]; [3: high]. (iii) Scattered: [0: not present]; [1: <10%]; [2: 10-50%]; [3: >50%]. T cell (CD3): ‘Clusters’ and ‘Scattered’ as above for B cells. Macrophages: [0: negative]; [1: <10% low]; [2: 10-50% medium]; [3: >50% high] [28]. Synovia were classified into 3 pre-defined histopathological subtypes: (1) Follicular: predominantly lymphoid with ELS; (2) Diffuse: dominated by myeloid cells and T lymphocytes; and (3) Pauci-immune: fibroblast dominated, with minimal inflammatory infiltrate [36, 37]. **Fig. S3.** Western Blot for isoform specific antibodies. **A** Western blot using the anti-p53β antibody 79.3 (sourced from JC Bourdon lab) on total protein of PC-3 cells transfected with either control plasmid (Empty Vector) or a plasmid expressing either Δ133p53α, Δ133p53β or Δ133p53γ isoforms respectively. **B** Western blot using KJCA133, an anti-Δ133p53 antibody (sourced from JC Bourdon lab) on total protein of PC-3 cells transfected with either control plasmid (Empty Vector) or a plasmid expressing Δ133p53γ. **Fig. S4**. Distribution of mRNA expression levels of *FL/Δ40TP53_T1*, *FL/Δ40TP53_T2*, *Δ133TP53*, *TP53α*, *TP53β *in OA and RA synovial tissue. Dots represent data from individuals in each group. Lines represent mean and SD. Significance was determined using unpaired t-test with Welch’s correction. *p < 0.05, **p < 0.01, ***p < 0.001. **Fig. S5**. p53β protein co-localises with CD55^+^ fibroblast-like synoviocytes (FLS), CD68^+^ macrophage-like synoviocytes (MLS) and macrophages, and CD90^+^ cells. Panels 1 through to 4 describe panels left to right. **A** FLS are CD55^+^; MLS and macrophages (M) are CD68^+^ **B** p53β expressing cells (green, panel 1), CD55+/ CD68+ expressing cells (red, panel 2), Merged (panel 3) showing co-localisation of p53β^+^ with either CD55+ or CD68+ cells (yellow) and nuclei (blue). 400x magnification; scale bar 50µm. **C** p53β is highly expressed in CD90^+^ cells surrounding ELS (top row). p53β is expressed in cells that are CD90^+^ that resemble plasma cells (middle row, panel 1; arrowed) and adjacent to p53β^+^ endothelium (middle row, panel 2) and in endothelial cells (middle row, panel 3). CD90^+^ and vWF^+^ cells in endothelial and sub-endothelial layer (bottom row) **D** The percentage of CD55^+^, CD68^+^, and CD90^+^ cells that also co-express p53β. Each dot represents results from each individual image field and a minimum of 100 cells. The lines represent the mean and SD. Significance was determined using unpaired t test with Welch’s correction (***p < 0.001; ****p <0.0001). CD90^+^/p53β^+^ vs CD68^+^/p53β^+^, *p*<0.0001; CD90^+^/p53β^+^ vs CD55^+^/p53β^+^, *p*<0.0001; CD68+/p53β^+^ vs CD55^+^ p53β^+^, *p*<0.0004. **Fig. S6. **MCP-1, MIP-1a, MIP-1b and RANTES were elevated in the serum from Δ122p53+/- (blue) compared to p53+/- (red) mice. The bars represent the concentration of the individual cytokines from 4 pooled serum samples. The error bars represent 97% CI. Significance was determined using unpaired t-test with Welch’s correction; *p < 0.05.

**Additional file 2:** **Table S1.** Correlations of Synovial *TP53* Transcript Expression, Select Gene Expression and Plasma Cytokine Measures. Shown are Spearman correlation r values between transcript levels of various p53 isoforms and select inflammatory genes present in synovial tissue and with measures of plasma cytokine. Significant correlations are shown in bold with asterisks indicating level of significance: * *p*<0.05; ** *p*<0.01; ***p<0.001; and ****p<0.0001.

## Data Availability

Clinical and demographic characteristics of the rheumatoid arthritis and osteoarthritis cohorts are detailed in Table 1.

## Abbreviations

RA: Rheumatoid arthritis
ELS: Ectopic lymphoid structures
Wt: Wild-type
FLp53: Full-length p53
RT-qPCR: Real-time quantitative reverse transcription PCR
ddPCR: Droplet digital PCR
FFPE: Formalin-fixed paraffin-embedded
