# Supplementary material for: Activated CD90/Thy-1 fibroblasts co-express the Δ133p53β isoform and are associated with highly inflamed rheumatoid arthritis
Source: Arthritis Res Ther. 2023 Apr 15;25:62. doi: 10.1186/s13075-023-03040-8 (PMC10105423; doi:10.1186/s13075-023-03040-8)
Supplement: Supplementary file 1 — Additional file 1: Fig. S1. Schematic of TP53 transcripts and protein isoforms. A Top panel shows the gene structure of TP53. The bottom panel shows the 9 TP53 transcripts resulting from alternative splicing (α, β, and γ) and alternative promoter usage (P1 and P2). RT-qPCR regions are shown as a red arrow that correspond to the 5’ end of the TP53 transcript for FL/Δ40TP53_T1, FL/Δ40TP53_T2, Δ133TP53, and those to the 3’ end for TP53α, TP53β and TP53γ. Light blue region represents the coding exons; grey regions represent the untranslated regions. B Region recognized by the rabbit polyclonal antibody KJCA133αβγ and 79.3 designed to specifically detect the Δ133p53 and TP53β isoform families respectively. Fig. S2. Classification of RA subtype based on IHC. Immunoscores were assigned based on the following criteria: (i) B cell (CD20): Follicles: [0: none evident]; [1: <5]; [2: 5-10]; [3: >10]. (ii) Clusters: [0: negative]; [1: low]; [2: medium]; [3: high]. (iii) Scattered: [0: not present]; [1: <10%]; [2: 10-50%]; [3: >50%]. T cell (CD3): ‘Clusters’ and ‘Scattered’ as above for B cells. Macrophages: [0: negative]; [1: <10% low]; [2: 10-50% medium]; [3: >50% high] [28]. Synovia were classified into 3 pre-defined histopathological subtypes: (1) Follicular: predominantly lymphoid with ELS; (2) Diffuse: dominated by myeloid cells and T lymphocytes; and (3) Pauci-immune: fibroblast dominated, with minimal inflammatory infiltrate [36, 37]. Fig. S3. Western Blot for isoform specific antibodies. A Western blot using the anti-p53β antibody 79.3 (sourced from JC Bourdon lab) on total protein of PC-3 cells transfected with either control plasmid (Empty Vector) or a plasmid expressing either Δ133p53α, Δ133p53β or Δ133p53γ isoforms respectively. B Western blot using KJCA133, an anti-Δ133p53 antibody (sourced from JC Bourdon lab) on total protein of PC-3 cells transfected with either control plasmid (Empty Vector) or a plasmid expressing Δ133p53γ. Fig. S4. Distribution of mRNA express [file 13075_2023_3040_MOESM1_ESM.pdf]

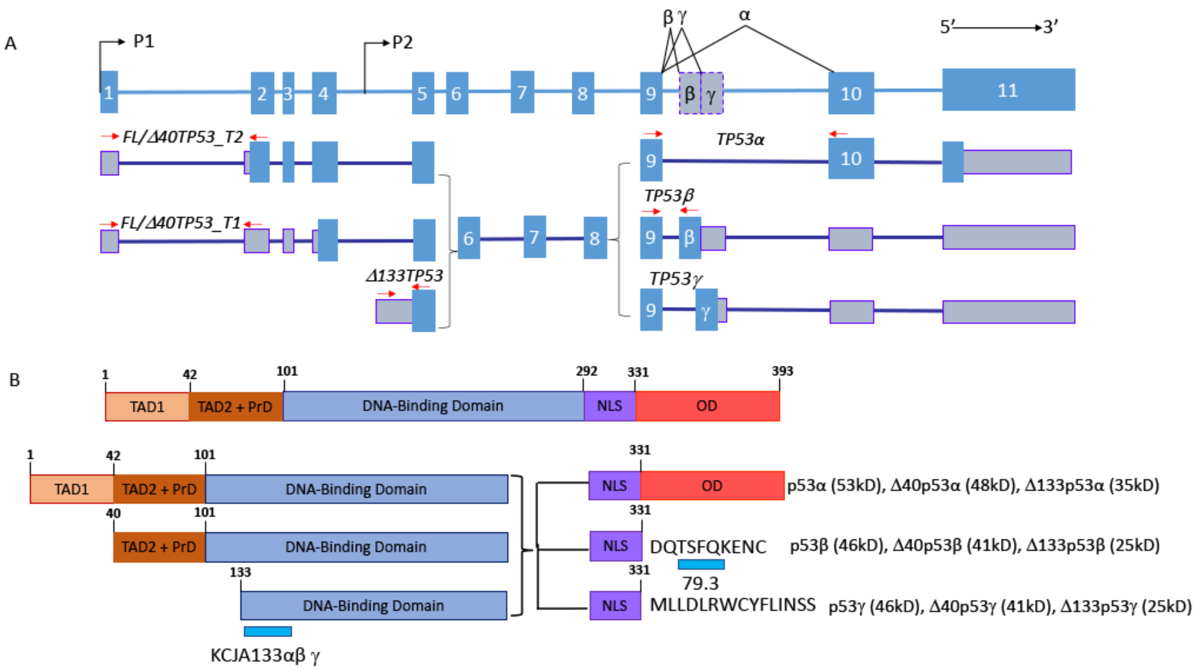

Supplementary Figure 1. Schematic of *TP53* transcripts and protein isoforms. A Top panel shows the gene structure of *TP53*. The bottom panel shows the 9 *TP53* transcripts resulting from alternative splicing (α, β, and γ) and alternative promoter usage (P1 and P2). RT-qPCR regions are shown as a red arrow that correspond to the 5' end of the *TP53* transcript for *FL/Δ40TP53_T1*, *FL/Δ40TP53_T2*, *Δ133TP53,* and those to the 3' end for *TP53*α, *TP53*β and *TP53*γ. Light blue region represents the coding exons; gray regions represent the untranslated regions. **B** Region recognized by the rabbit polyclonal antibody KCJA133αβγ and 79.3 designed to specifically detect the Δ133p53 and *TP53*β isoform families respectively.

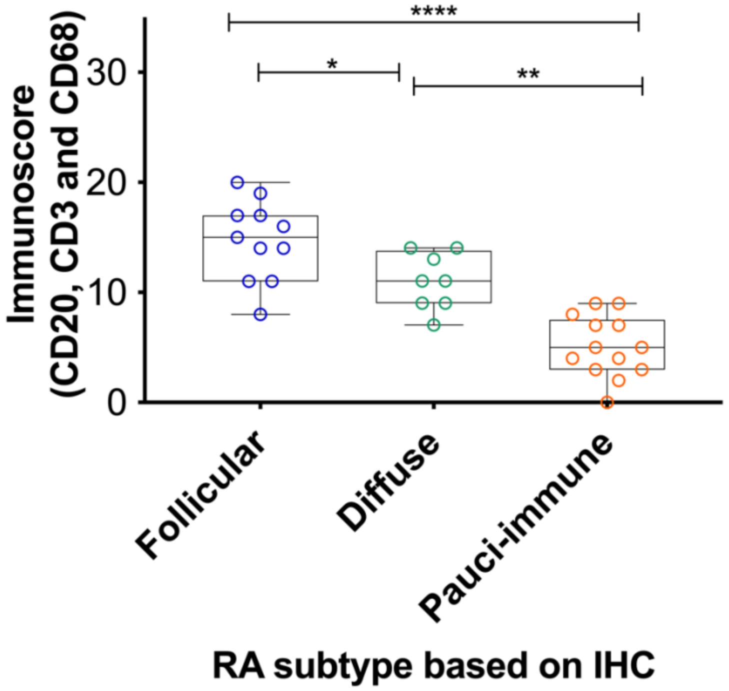

Supplementary Figure 2. Classification of RA subtype based on IHC.

Immunoscores were assigned based on the following criteria: (i) B cell (CD20): Follicles: [0: none evident]; [1: <5]; [2: 5-10]; [3: >10]. (ii) Clusters: [0: negative]; [1: low]; [2: medium]; [3: high]. (iii) Scattered: [0: not present]; [1: <10%]; [2: 10-50%]; [3: >50%]. T cell (CD3): 'Clusters' and 'Scattered' as above for B cells. Macrophages: [0: negative]; [1: <10% low]; [2: 10-50% medium]; [3: >50% high] [28]. Synovia were classified into 3 pre-defined histopathological subtypes: (1) Follicular: predominantly lymphoid with ELS; (2) Diffuse: dominated by myeloid cells and T lymphocytes; and (3) Pauci-immune: fibroblast dominated, with minimal inflammatory infiltrate (36, 37).

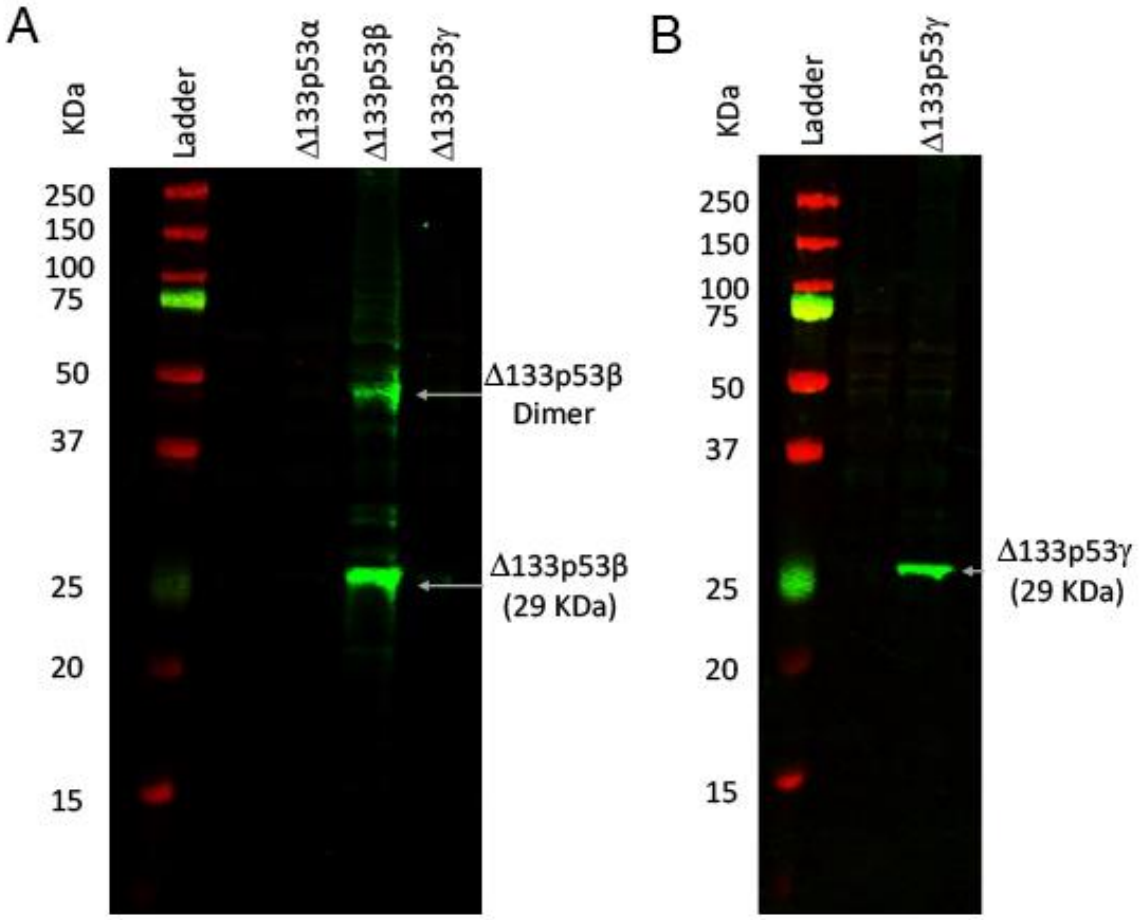

Supplementary Figure 3. Western Blot for isoform specific antibodies. A Western blot using the anti-p53β antibody 79.3 (sourced from JC Bourdon lab) on total protein of PC-3 cells transfected with either control plasmid (Empty Vector) or a plasmid expressing either Δ133p53α, Δ133p53β or Δ133p53γ isoforms respectively. **B** Western blot using KJCA133, an anti-Δ133p53 antibody (sourced from JC Bourdon lab) on total protein of PC-3 cells transfected with either control plasmid (Empty Vector) or a plasmid expressing Δ133p53γ.

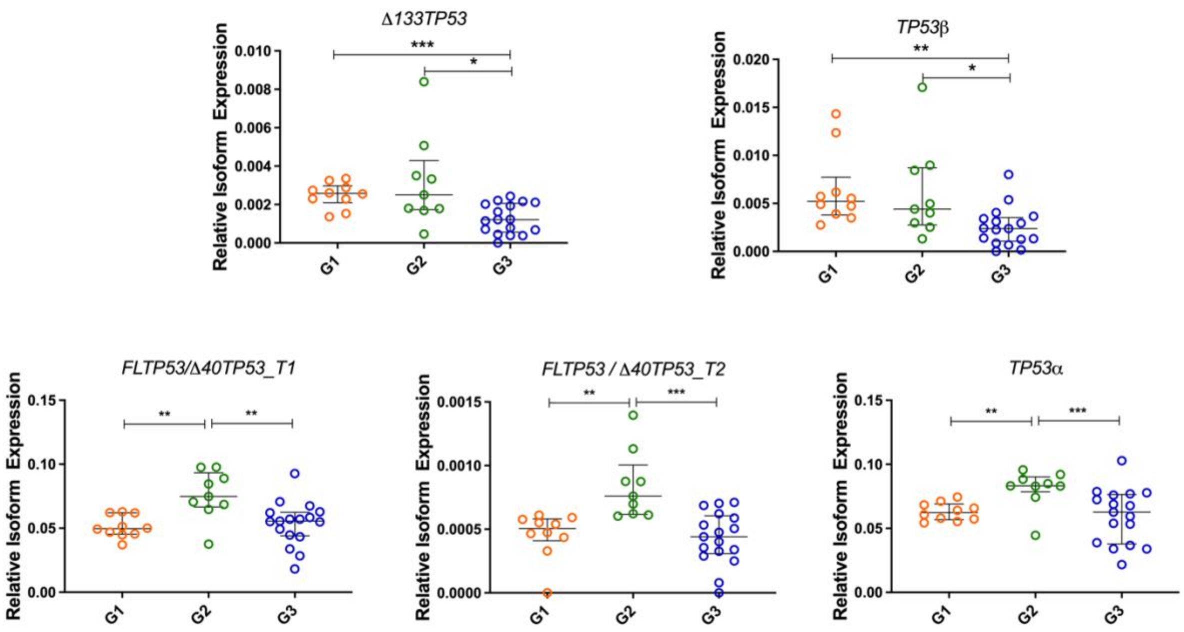

Supplementary Figure 4. Distribution of mRNA expression levels of *FL/Δ40TP53_T1*, *FL/Δ40TP53_T2*, *Δ133TP53*, *TP53α*, *TP53β* in OA and RA synovial tissue.

Dots represent data from individuals in each group. Lines represent mean and SD. Significance was determined using unpaired t-test with Welch's correction. *$p < 0.05$, **$p < 0.01$, ***$p < 0.001$.

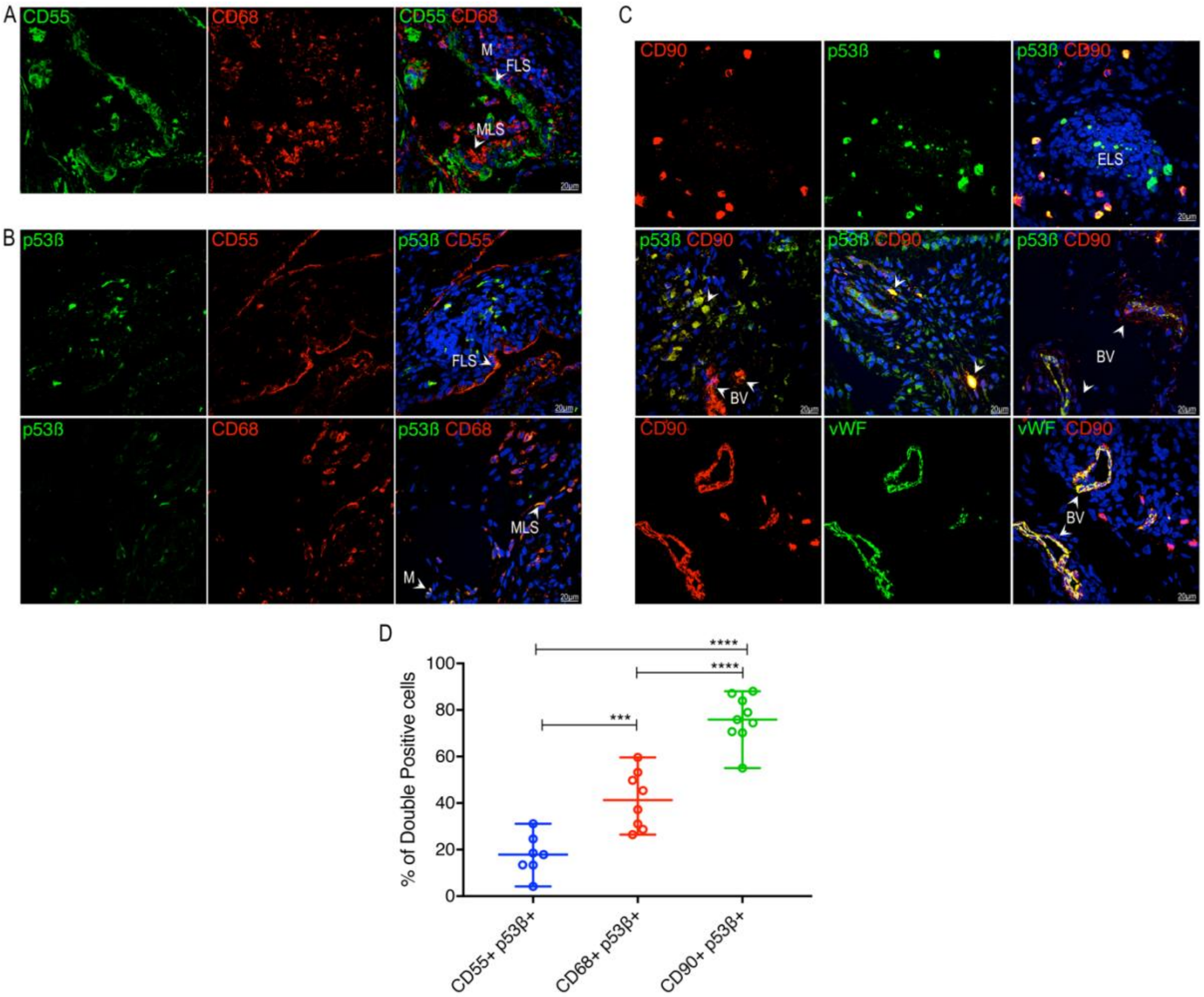

Supplementary Figure 5. **p53β protein co-localises with CD55$^+$ fibroblast-like synoviocytes (FLS), CD68$^+$ macrophage-like synoviocytes (MLS) and macrophages, and CD90$^+$ cells**.

Panels 1 through to 4 describe panels left to right. **A** FLS are CD55$^+$; MLS and macrophages (M) are CD68$^+$ **B** p53β expressing cells (green, panel 1), CD55+/ CD68+ expressing cells (red, panel 2), Merged (panel 3) showing co-localisation of p53β$^+$ with either CD55+ or CD68+ cells (yellow) and nuclei (blue). 400x magnification; scale bar 50µm. **C** p53β is highly expressed in CD90$^+$ cells surrounding ELS (top row). p53β is expressed in cells that are CD90$^+$ that resemble plasma cells (middle row, panel 1; arrowed) and adjacent to p53β$^+$ endothelium (middle row, panel 2) and in endothelial cells (middle row, panel 3). CD90$^+$ and vWF$^+$ cells in endothelial and sub-endothelial layer (bottom row) **D** The percentage of CD55$^+$, CD68$^+$, and CD90$^+$ cells that also co-express p53β. Each dot represents results from each individual image field and a minimum of 100 cells. The lines represent the mean and SD. Significance was determined using unpaired t test with Welch's correction (***$p < 0.001$; ****$p < 0.0001$). CD90$^+$/p53β$^+$ vs CD68$^+$/p53β$^+$, $p<0.0001$; CD90$^+$/p53β$^+$ vs CD55$^+$/p53β$^+$, $p<0.0001$; CD68+/p53β$^+$ vs CD55$^+$ p53β$^+$, $p<0.0004$.

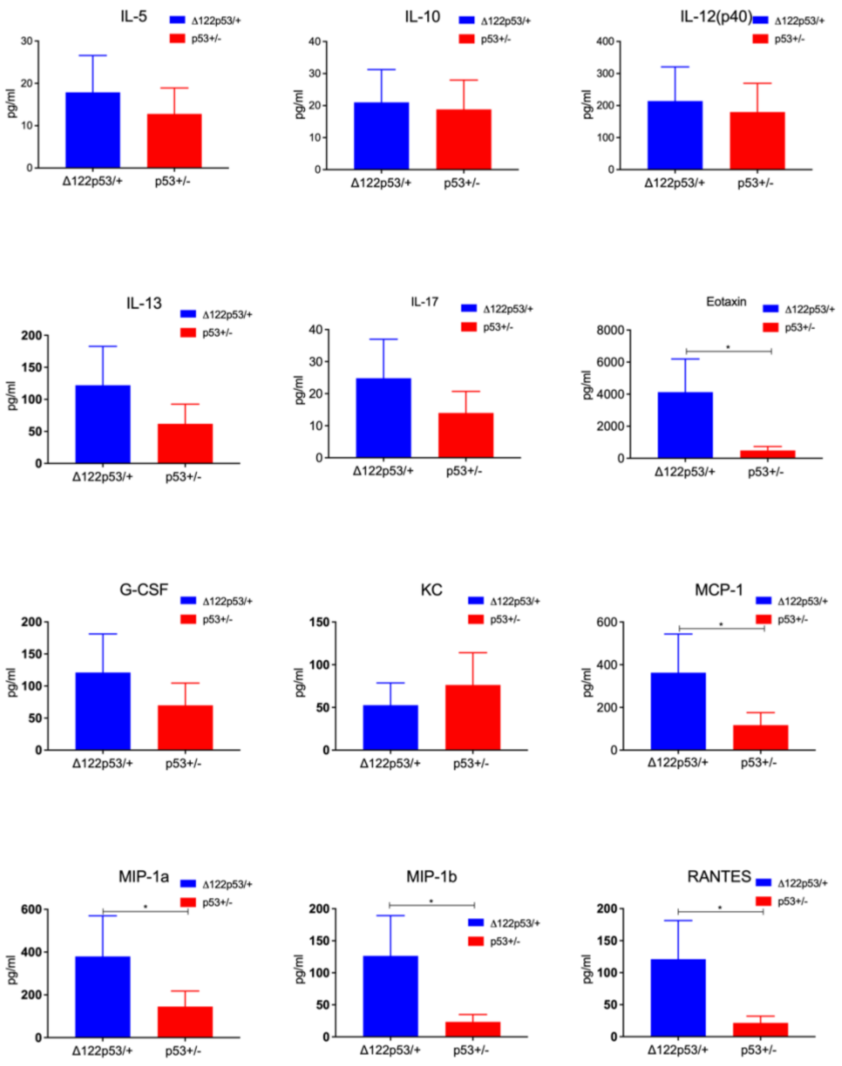

Supplementary Figure 6. MCP-1, MIP-1a, MIP-1b and RANTES were elevated in the serum from Δ122p53+/- (blue) compared to p53+/- (red) mice.

The bars represent the concentration of the individual cytokines from 4 pooled serum samples. The error bars represent 97% CI. Significance was determined using unpaired t-test with Welch’s correction; *$p < 0.05$.
