# Supplementary material for: Activated CD90/Thy-1 fibroblasts co-express the Δ133p53β isoform and are associated with highly inflamed rheumatoid arthritis
Source: Arthritis Res Ther. 2023 Apr 15;25:62. doi: 10.1186/s13075-023-03040-8 (PMC10105423; doi:10.1186/s13075-023-03040-8)
Supplement: Supplementary file 2 — Additional file 2: Table S1. Correlations of Synovial TP53 Transcript Expression, Select Gene Expression and Plasma Cytokine Measures. Shown are Spearman correlation r values between transcript levels of various p53 isoforms and select inflammatory genes present in synovial tissue and with measures of plasma cytokine. Significant correlations are shown in bold with asterisks indicating level of significance: * p<0.05; ** p<0.01; ***p<0.001; and ****p<0.0001. [file 13075_2023_3040_MOESM2_ESM.docx]

**Supplementary Table 1.** Correlations of Synovial *TP53* Transcript Expression, Select Gene Expression and Plasma Cytokine Measures.

|  |  | Synovial Tissue *TP53* Transcripts | | | | | | | | | | | | |  |
| --- | --- | --- | --- | --- | --- | --- | --- | --- | --- | --- | --- | --- | --- | --- | --- |
| Synovial Tissue Transcript/Gene | Isoform/  Gene | *FL/Δ40TP53_T1* |  | | |  | | |  | | |  | | | |
|  |  |  | *FL/Δ40TP53_T2* | | |  | | |  | | |  | | | |
|  | *Δ40p53* | **0.648**^***^ |  | | TP53α | |  | | |  | | |  |  |  |
|  | *TP53a* | **0.587**^***^ | **0.861**^****^ | |  | | *Δ133TP53* | | |  | | |  |  |  |
|  | *Δ133p53* | **0.341**^*^ | 0.231 | | **0.346**^*^ | |  | | | TP53β | | |  |  |  |
|  | *TP53β* | 0.195 | 0.294 | | **0.443**^*^ | | **0.543**^*^ | | |  | | |  |  |  |
|  | *CD21L* | 0.257 | 0.248 | | 0.243 | | 0.285 | | | 0.078 | | |  |  |  |
|  | *IL17A* | 0.055 | 0.028 | | 0.024 | | 0.030 | | | 0.109 | | |  |  |  |
|  | *IL6* | 0.131 | -0.222 | | -0.062 | | 0.061 | | | -0.067 | | |  |  |  |
|  | *TNFA* | 0.025 | -0.095 | | -0.262 | | 0.003 | | | 0.226 | | |  |  |  |
|  | *IL27A* | -0.130 | -0.221 | | -0.238 | | 0.162 | | | 0.030 | | |  |  |  |
|  | *IL27RA* | 0.009 | 0.044 | | -0.012 | | 0.295 | | | 0.264 | | |  |  |  |
|  | *IL27B* | 0.062 | 0.018 | | -0.003 | | **0.536**^*^ | | | **0.543**^*^ | | |  |  |  |
|  | *IL27RB* | 0.176 | 0.379 | | 0.312 | | 0.379 | | | 0.489 | | |  |  |  |
|  | *JAG1* | **0.478**^*^ | **0.583**^***^ | | **0.621**^**^ | | -0.108 | | | 0.223 | | |  |  |  |
|  | *NOTCH3* | 0.293 | **0.507**^*^ | | **0.617**^**^ | | 0.023 | | | 0.248 | | |  |  |  |
|  |  |  |  | | |  | | |  | | |  | | | |
| Plasma Cytokine Measure |  |  |  |  | | | |  | | |  | | |  |  |
|  | IL-1β | 0.340 | **0.424**^*^ | **0.585**^**^ | | | | 0.086 | | | 0.160 | | |  |  |
|  | IL-4 | 0.237 | 0.369 | **0.521**^*^ | | | | **0.674**^**^ | | | **0.443*** | | |  |  |
|  | IL-6 | 0.399 | 0.368 | **0.464**^*^ | | | | 0.133 | | | **0.443**^*^ | | |  |  |
|  | IL-10 | **0.482**^*^ | **0.423**^*^ | **0.589**^**^ | | | | 0.414 | | | 0.311 | | |  |  |
|  | IL-17A | 0.246 | 0.217 | 0.324 | | | | 0.301 | | | 0.253 | | |  |  |
|  | TNF-α | 0.227 | 0.113 | 0.361 | | | | **0.506**^*^ | | | 0.371 | | |  |  |
|  | IL-22 | 0.358 | 0.013 | 0.201 | | | | 0.295 | | | 0.008 | | |  |  |
|  | IL-23 | **0.615**^**^ | **0.446**^*^ | **0.602**^**^ | | | | 0.302 | | | 0.183 | | |  |  |
|  | IFN-γ | 0.385 | 0.156 | 0.130 | | | | -0.188 | | | -0.361 | | |  |  |
|  | IL-25 | **0.682**^***^ | 0.372 | 0.390 | | | | -0.026 | | | 0.012 | | |  |  |
|  | IL-31 | **0.472**^*^ | 0.160 | 0.337 | | | | -0.149 | | | -0.253 | | |  |  |
|  | IL-33 | 0.071 | 0.205 | **0.434**^*^ | | | | **0.540*** | | | **0.468*** | | |  |  |
|  | sCD40L | 0.084 | 0.206 | 0.378 | | | | 0.175 | | | 0.185 | | |  |  |
|  | IL-17F | **0.658**^**^ | **0.413**^*^ | **0.516**^*^ | | | | 0.271 | | | 0.111 | | |  |  |
|  | IL-21 | **0.651**^*^ | 0.243 | 0.395 | | | | 0.166 | | | 0.147 | | |  |  |
|  |  |  |  | | |  | | |  | | |  | | | |

Shown are Spearman correlation r values between transcript levels of various p53 isoforms and select inflammatory genes present in synovial tissue and with measures of plasma cytokine. Significant correlations are shown in bold with asterisks indicating level of significance: * p<0.05; ** p<0.01; ***p<0.001; and ****p<0.0001
